# Precocious Puberty and the Lin28/Let7 Pathway: The Therapeutic Effect of the Nourishing “Yin” and Purging “Fire” Traditional Chinese Medicine Mixture in a Rat Model

**DOI:** 10.1155/2018/4868045

**Published:** 2018-06-26

**Authors:** Yuanyuan He, Xinghui Han, Wen Sun, Jian Yu, Amin Tamadon

**Affiliations:** Department of Integrative Medicine, Children's Hospital of Fudan University, Shanghai 200032, China

## Abstract

The present study aims to investigate the effects of the nourishing “Yin” and purging “Fire” Traditional Chinese Medicine (TCM) herb mixture on precocious puberty and TCM may act through hypothalamic Lin28/let7 pathway expression in the precocious puberty model rats. Meanwhile, to confirm the relationship between Lin28/let7 pathway and puberty by overexpression* Lin28a,* in the first part of this study, female rats were randomly allocated into untreated controls, the precocious puberty (PP) model group, the PP control group, and the PP + TCM group. Rats on postnatal day 5 were injected danazol to establish the PP model. From days 15 to 35, the rats in the TCM group were given the TCM twice daily. Vaginal opening, sex-related hormones, and body and reproductive organ weights were measured, and the expressions of hypothalamic* Lin28a *and* Lin28b* mRNA and* let7a* and* let7b *miRNA were detected. In addition, in the second part, the effects of overexpression of* Lin28a* on the vaginal opening time were evaluated. In the two parts of the study, we found that, at the onset of puberty, a decrease in ovary weight, an increase in the serum levels of luteinizing hormone and progesterone, and increased expression levels of hypothalamic* Lin28b* mRNA were observed in the PP + TCM group compared to the PP model group. The vaginal opening time was significantly delayed upon overexpression of* Lin28a*. Above all, the mechanism by which the TCM treats precocious puberty is thus likely to be associated with inhibition of the hypothalamic Lin28/let7 signaling pathway and our findings provide in-depth insight into the relationship between the overexpression of* Lin28a* gene in the hypothalamus and the onset of puberty.

## 1. Introduction

There has been increased interest in the study of the age of puberty onset in girls [1, 2], and there is evidence that the prevalence of idiopathic central precocious puberty has increased [3]. Although the mechanism behind precocious puberty is still not clear, there is evidence to suggest that genetic modulation of the onset of puberty might occur from the additive effect of different genes and pathways [4–6]. The onset of puberty is a complex biological process involving numerous factors under the control of the neuroendocrine pathways that are regulated as part of the hypothalamus-pituitary-gonadal (HPG) axis [7].

The key step in puberty onset is activation of gonadotropin releasing hormone (GnRH) pulses and secretion [8]. At the onset of puberty, GnRH stimulates the secretion of the pituitary gonadotropins luteinizing hormone (LH) and follicle-stimulating hormone (FSH). LH and FSH in turn stimulate the ovaries to initiate follicular growth and luteal formation that secretes the sex steroid hormones estrogen (E2) and progesterone (P4).

The role of the Lin28/let7 signal pathway in pubertal development is well studied. Lin28 (Lin28a) is a highly conserved RNA-binding protein that was first identified in* Caenorhabditis elegans* and was shown to be involved in the regulation of* C. elegans* development [9]. The let7 family of microRNAs (miRNAs), as classic representatives of miRNAs, were also initially identified in* C. elegans*, and mutations in the* let7* gene lead to overall abnormalities in body development [10]. A previous study showed that let7 can decrease the expression of Lin28 by inhibiting the translation of Lin28 after transcription [11]. At the same time, Lin28 can inhibit the maturation of let7 through multiple mechanisms [12, 13]. Therefore, there appears to be a double-negative feedback loop in the Lin28/let7 signaling pathway.

In mammals, two Lin28-related genes have been identified, referred to as* Lin28a* (also known as* Lin28*) and* Lin28b* [14–16]. A transgenic mouse model that overexpresses* Lin28a* has been established, and female mice in this model have delayed vaginal opening time compared with normal littermates. Both the time of the first estrus and the age of the first pregnancy were also delayed, indicating that overexpression of* Lin28a* in mice can delay pubertal development [17]. In S. Sangiao research, changes in the* c-Myc/Lin28b/let7* pathway were detected in models of delayed puberty, and in this study they also found the changes in the expression of the* Lin28/let7* axis in the rat hypothalamus during the postnatal maturation and after different manipulations that disturb puberty [18], while, in the studies of Grieco et al. and Corre et al. [19, 20], they showed a complex system of regulation by* Lin28a and Lin28b and let7a and let7b, *raising the possibility that this pathway may contribute to the growth and puberty in humans. Meanwhile, 32 single-nucleotide polymorphisms associated with age at menarche have been found in human genome-wide association studies. Closely related to age at menarche, in 2009, they found these 32 SNPs with the most significantly associated SNP being at the* LIN28B* locus [21–23]. During 2008 to 2012, a meta-analysis of the available data has validated these observations and shown that there is a close correlation between the expression of* LIN28B* and body mass index, breast development, and adult height [24, 25]. In 2014, they identified 697 variants at genome-wide significance that together explain one-fifth of heritability for adult height [26]. Furthermore, mutations in the* LIN28B* gene have been shown to lead to precocious puberty in girls [27]. In addition, based on the most recent study, there are over 380 loci associated with age at menarche concluding most of SNPs with the lowest P value, or most significantly associated which are at the* LIN28B l*ocus [28]. Until now, there were several new findings in the genome-wide association studies about* LIN28B.* In these studies Coignet et al. showed the effects of* LIN28B* are various and connective with postnatal growth, pubertal growth, final height, cancer risk, and even more possibilities [29–36]. With the fact that genome-wide association studies have identified genetic variations in or near the* LIN28B* gene as associated with age at menarche, to evaluate the association of the four loci in or near* LIN28B* with ICPP in Chinese girls, Hu et al. found that two of common genetic variations of* LIN28B* may contribute to ICPP susceptibility in Chinese girls [37].

These studies described above suggest that the Lin28/let7 signaling pathway might regulate pubertal development through the HPG axis. However, the mechanism through which the Lin28/let7 signaling pathway regulates the HPG axis and controls the initiation of puberty is still not clear, and it is still not known whether the Lin28/let7 signaling pathway is associated with the pathogenesis of precocious puberty.

Positive effects of the nourishing “Yin” and purging “Fire” Traditional Chinese Medicine (TCM) mixture on precocious puberty have been demonstrated in girls [38]. The TCM mixture is an original prescription from our Department of Integrative Medicine, Children's Hospital of Fudan University (Shanghai pharmacists system number Z05170908). The mixture mainly consists of* Rehmannia glutinosa* (Sheng-Di-Huang),* Scrophularia buergeriana* (Xuan-Shen),* Anemarrhena asphodeloides* (Zhi-Mu),* Cortex Phellodendri* (Huang-Bai), and so forth (Table 1) [39, 40]. This TCM mixture delays the timing of advanced puberty and the development of secondary sexual characteristics not only in humans of the clinic research, but also in the precocious puberty rat model in our previous studies [4, 5, 40–42], and it has been shown to retard the growth of the ovaries and uterus [38, 43, 44] and to prevent the shortened final adult stature caused by precocious puberty [45]. The nourishing “Yin” and purging “Fire” TCM herb mixture downregulates the expression of GnRH and delays the development of puberty in a rat model of precocious puberty [46].

Based on the results from these experiments, the relationship between the Lin28/let7 signaling pathway and the pathogenesis of precocious puberty was studied in the danazol-induced precocious puberty model in female rats. Recent evidence suggests that the Lin28/let7 pathway might be a critical regulator of GnRH release and that it might play an important role in regulating the onset of puberty [47]. The aim of the present study was to determine the effects of the nourishing “Yin” and purging “Fire” TCM mixture on the hypothalamic Lin28/let7 pathway in female precocious puberty rats and to determine the effect of overexpression of* Lin28a* on puberty onset. To investigate the effect of overexpression of* Lin28a* on puberty onset, the* Lin28a* gene was incorporated into a lentiviral vector that was then injected by stereotaxic methods into the hypothalamic arcuate nucleus (ARC). The timing of vaginal opening and reproductive tissue maturation was evaluated along with measurements of serum sex steroid hormone levels. Our study is the first to explore the effects of the TCM mixture on the hypothalamus Lin28/let7 signaling pathway, aiming to elucidate the mechanism by which this TCM mixture delays advanced puberty onset of precocious puberty.

## 2. Materials and Methods

### 2.1. Animals

Female Sprague-Dawley rats were purchased from the Jiesijie Animal Center (Shanghai, China, license number: SCXK (Shanghai) 2012-0002), and 3-day-old rats and their mothers were used in Experiment 1 and 20-day-old rats were used in Experiment 2. Animals were housed in the facilities of the Department of Neurobiology and Integrative Medicine of Fudan University and had free access to food and water with controlled ambient temperature (24 ± 2°C) and humidity (67 ± 1.5%) with a 12 h/12 h (light/dark) schedule in a room shielded from outside noise. All procedures were approved by the Fudan University Animal Care and Use Committee in accordance with the National Institutes of Health Guide for the Care and Use of Laboratory Animals (No. [2014]059; 25/2/2014).

### 2.2. Experiment 1: TCM Treatment of Precocious Puberty and Its Effect on Hypothalamic Lin28/Let7 Expression 

#### 2.2.1. Preparation of the Nourishing “Yin” and Purging “Fire” TCM Mixture

The nourishing “Yin” and purging “Fire” TCM mixture is an original prescription developed by the Department of Integrative Medicine, Children's Hospital of Fudan University (Shanghai pharmacists system number Z05170908, Shanghai, China) (the ingredients of the formula are showed in Table 1) based on a traditional formula of nourishing Kidney-Yin to remove ministerial fire for treatment of precocious puberty [39, 40]. According to the law of Syndrome Differentiation of TCM, female precocious puberty is the result of Kidney-Yin deficiency and Kidney-Yang exuberance [41]. Therefore, to remove this effect various traditional formulations are available in TCM for treatment of precocious puberty [42].

To achieve the exact traditional formula, the mixture was prepared using the traditional water-extraction/alcohol-precipitation method [4] using a thermostat electric set (Zhengzhou Great Wall Scientific Industrial & Trade Co. Ltd., Zhengzhou, China) to decoct the crude drugs for 40 min at 100°C. The thermostat electric set was refilled with water for decocting for another 40 min. The extracted liquid was then collected and concentrated on a rotary evaporator (Buchi, Switzerland) (15 min at 4°C at 1,000 rpm/min). Following this, absolute ethanol was slowly added to dilute the mixture to a final concentration of 60% ethanol, and the mixture was incubated at 4°C for 72 h. Finally, the ethanol was removed on the rotary evaporator (15 min at 4°C at 1,000 rpm/min), and the final drug was obtained at a final concentration of 2.7 g per ml.

#### 2.2.2. Experimental Groups and the Induction of Precocious Puberty Models

**Figure 1:**
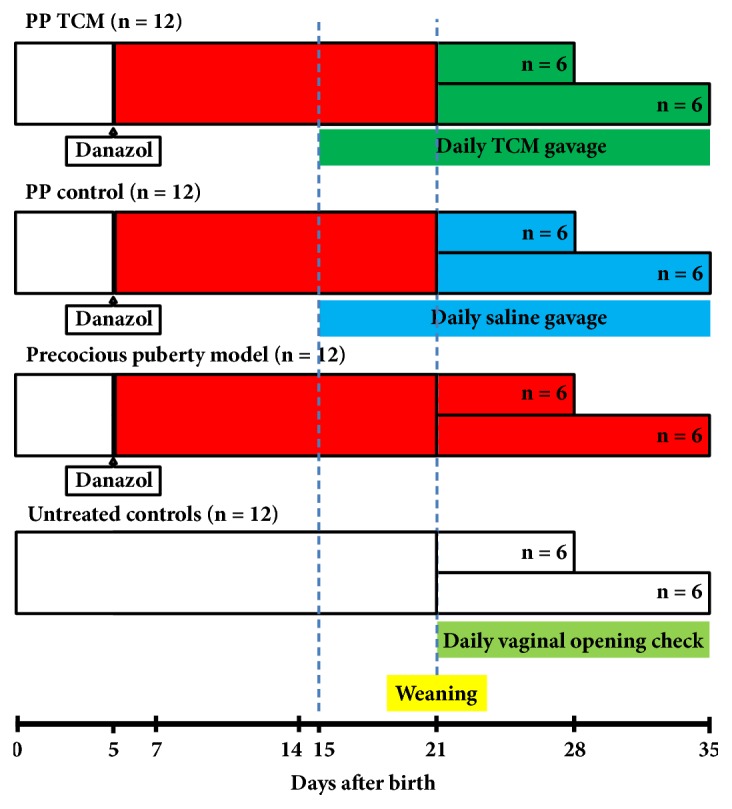
Schematic diagram of the experimental design of Experiment 1, including precocious puberty model induction, TCM treatment, puberty check, and sampling time for evaluation of the effect of the nourishing “Yin” and purging “Fire” Chinese TCM mixture on a rat model of precocious puberty and on the Lin28/let7 pathway.

Female Sprague-Dawley rats aged 3 days were used to observe the day of vaginal opening, and these were divided into untreated controls (n = 12), the precocious puberty (PP) model group (n = 12), the PP + TCM treatment group (n = 12), and the PP control group (n = 12) (Figure 1). Precocious puberty was induced in the PP group, the PP + TCM treatment group, and the PP control group. The rats in the precocious puberty groups were subcutaneously injected at day 5 after birth with a single dose of 300 *μ*g of danazol (Hualian Pharm Ltd., Shanghai, China) dissolved in 25 *μ*l vehicle of propylene glycerol: ethanol (1:1, v/v) and allowed to grow without further treatment [48]. From days 15 to 35, the rats in the PP + TCM treatment group and the PP control group were given the TCM mixture or saline, respectively, by a gavage needle (1 mL/100 g body weight) every morning (8:00) and every evening (18:00). This dosage was equivalent to the smallest dosage that has been used in the clinic for the treatment of precocious puberty. The rats were randomly sacrificed on day 28 (puberty onset, defined as when the vaginal opening was completed in the PP model group, n = 6) or on day 35 (puberty, n = 6).

#### 2.2.3. Samplings

Rats were weighed and anesthetized by intraperitoneal injection of 2% chloral hydrate (0.4 mL/100 g) before being sacrificed. Blood samples were collected from the jugular vein. Blood serum was then separated using a high-speed freezing centrifuge (Heraeus, Germany) and stored at −80°C until being assayed. To evaluate the hypothalamic expressions of* Lin28a* and* Lin28b* mRNA and* let7a* and* let7b* miRNA, hypothalamic samples were collected and stored at −80°C. The uteruses and ovaries were immediately dissected out of the surrounding fat by opening the abdominal cavity and were weighed to evaluate the organ coefficients according to the organ index formula ([organ wet weight (g)/body weight (g)] × 10^−4^) [49].

### 2.3. Experiment 2: Overexpression of Lin28a by Hypothalamic Lentivirus Injection

#### 2.3.1. The Construction of the Lentiviral Vector

The construction of the lentiviral vector was based on the Lin28a gene RefSeq number (NM_001109269), the sequence of which was obtained from the NCBI, and was prepared by Shanghai Genomics Co., Ltd. (Table 3). The pLOV-EF1a-PuroR-CMV-EGFP-P2A-3FLAG vector has a virus titer of 1.77 × 10^−9^ TU/ml (TU indicates the number of virus particles that can infect and enter the target cell population) and it encodes the EGFP-P2A-Lin28a-3FLAG protein. The lentivirus was stored in small aliquots at −80°C to avoid repeated freezing and thawing. The lentiviral vector was stored at 4°C and used within 1 week after the ice bath had melted.

#### 2.3.2. Determining the Location of the Hypothalamic ARC in Infant Rats

Following the method in* Rat Brain Stereotactic Mapping (Third Edition)* (Paxinos & Watson), lentiviral vector injections were simulated by slowly injecting 2 *μ*l trypan blue into the rat brain through a 2.5 *μ*l microinjection needle (Hamilton). After perfusion and after fixation, sucrose dehydration, and OCT embedding, the frozen sections of the brain were observed for the staining of trypan blue and the track of the injection needle. Based on this, the location of the hypothalamic ARC in the infant rats was defined as AP = −1.8 mm, Lat = −0.1 mm, and DV = −9.5 mm relative to the bregma.

#### 2.3.3. Groups and Lentiviral Vector Injection

**Figure 2:**
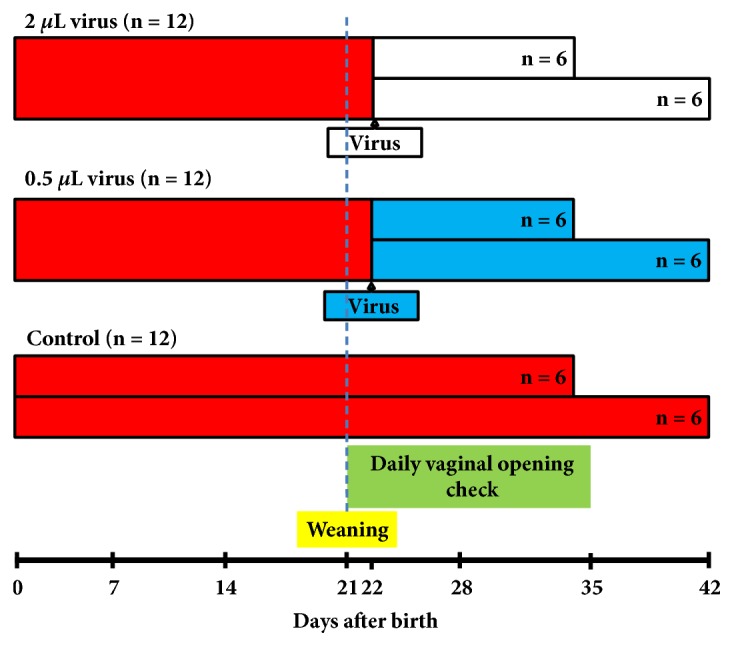
Schematic diagram of the design of Experiment 2, including lentivirus injection time, puberty check, and sampling time for evaluation of the effect of overexpression of Lin28a in the hypothalamus on puberty onset in rats.

Thirty-six female Sprague-Dawley rats at 20 days of age were randomly divided into the low-dose group (0.5 *μ*l lentiviral vector, n = 12), the high-dose group (2 *μ*l lentiviral vector, n = 12), and the control group (n = 12) (Figure 2). At day 22 after birth, the lentiviral vectors were microinjected into the right-side ARC of the rats at titers of 1.77 × 10^9^/ml using the stereotaxic method at a rate of 0.1 *μ*l/min and an injection time of 20 min. The microinjection needle (Hamilton) was gently extracted after 5 min following injection at a rate of 2 mm/min. The rats of the control group were injected slowly in the same location with 2 *μ*l negative control lentiviral vector carrying GFP. Daily inspections of vaginal opening and measurements of body weight were conducted. At day 35 (puberty) and day 46 (the postpubertal period), 6 rats in each group were sacrificed. Blood samples, hypothalamuses, uteruses, ovaries, and uterine tubes were collected. Serum hormones levels were measured using radioimmunoassay kits, and the expression of the* Lin28a* gene in the hypothalamus was measured by real-time PCR.

### 2.4. Hormone Level Detection

Blood samples were taken from the jugular vein of rats that had been anesthetized by intraperitoneal injection of 10% chloral hydrate (0.4 ml/100 g) 1 h after the last gavage (in Experiment 1) or after the vaginas of all rats had opened completely (in Experiment 2). The serum was then separated using a high-speed freezing centrifuge (15 min at −20°C at 3,000 rpm/min) (Heraeus, Germany) and stored at −80°C until being assayed. Serum LH, FSH, P4, and E2 levels were determined using radioimmunoassay kits (eBioscience, Usheshidu SA) according to the manufacturer's specifications. The sensitivities of the LH, FSH, P4, and E2 tests were 0.3 mIU/ml, 0.28 mIU/ml, 0.2 ng/l, and 1.7 pg/ml, respectively, and the intra-assay coefficients were 2.6%, 6%, 10%, and 4.5%, respectively.

### 2.5. Real-Time Reverse-Transcriptase Polymerase Chain Reaction (RT-PCR) Analysis

The expression of* Lin28a* and* Lin28b* mRNA in the hypothalamus of the rats was measured in triplicate by RT-PCR. Total RNA was isolated using the Direct-zol RNA Mini Prep Kit (Zymo Research Corp., USA), and RNA was reverse transcribed into cDNA using the 5× All-in-One RT Master Mix (ABM, Canada) (20 *μ*l volume) according to the manufacturer's protocols. The primers were synthesized by Shanghai Sangon Biotech Inc. (Shanghai, China) and are shown in Table 2. KAPA SYBR rapid quantitative PCR Master Mix (2×) (KAPA Biosystems Inc., USA) was added to 20 *μ*l of the reaction solution. The amplification conditions were as follows: predenaturation at 95°C for 3 min followed by denaturation at 95°C for 5 s, annealing at 60°C, and extension for 30 s with a total of 40 amplification cycles. The* Gapdh* gene was used as the internal standard using the 2^−ΔΔCt^ method to calculate the relative expression levels.

### 2.6. Statistical Analysis

Tissue weights, hormone concentrations, and mRNA and miRNA expressions with normal distributions (as determined by the Kolmogorov–Smirnov test) were analyzed by two-way ANOVA and the LSD* post hoc* test. Vaginal opening was analyzed by the Mann–Whitney* U* test (SPSS, version 22; Chicago, IL). The results are presented as the mean ± SEM, and p < 0.05 was considered significant.

## 3. Results

### 3.1. Experiment 1: The Effect of the TCM Mixture on Precocious Puberty and Hypothalamic Lin28/Let7 Expression

#### 3.1.1. The TCM Mixture Delayed Vaginal Opening in a Rat Model of Precocious Puberty

**Figure 3:**
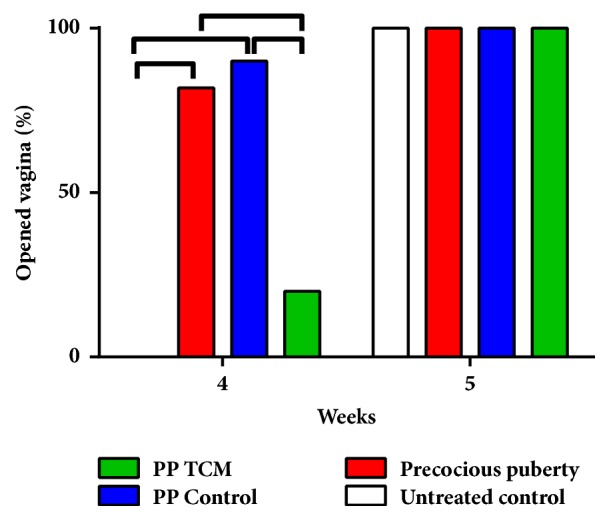
Effects of the nourishing “Yin” and purging “Fire” TCM mixture on the percent of vaginal opening in female rats (n = 6). Lines above the columns show significant differences between groups (p < 0.05).

The induction of precocious puberty increased the number of rats with a fully opened vagina on the day of puberty onset (p < 0.05, Figure 3). On the day of puberty onset, the number of rats with an opened vagina in the PP + TCM group was lower than in the PP model and PP control groups (p < 0.05) and was not different from the untreated controls (p > 0.05). After puberty, the number of rats with opened vagina was the same in each group (p > 0.05).

#### 3.1.2. The TCM Mixture Increased Body Weight and Decreased Ovary Weight in a Rat Model of Precocious Puberty

**Figure 4:**
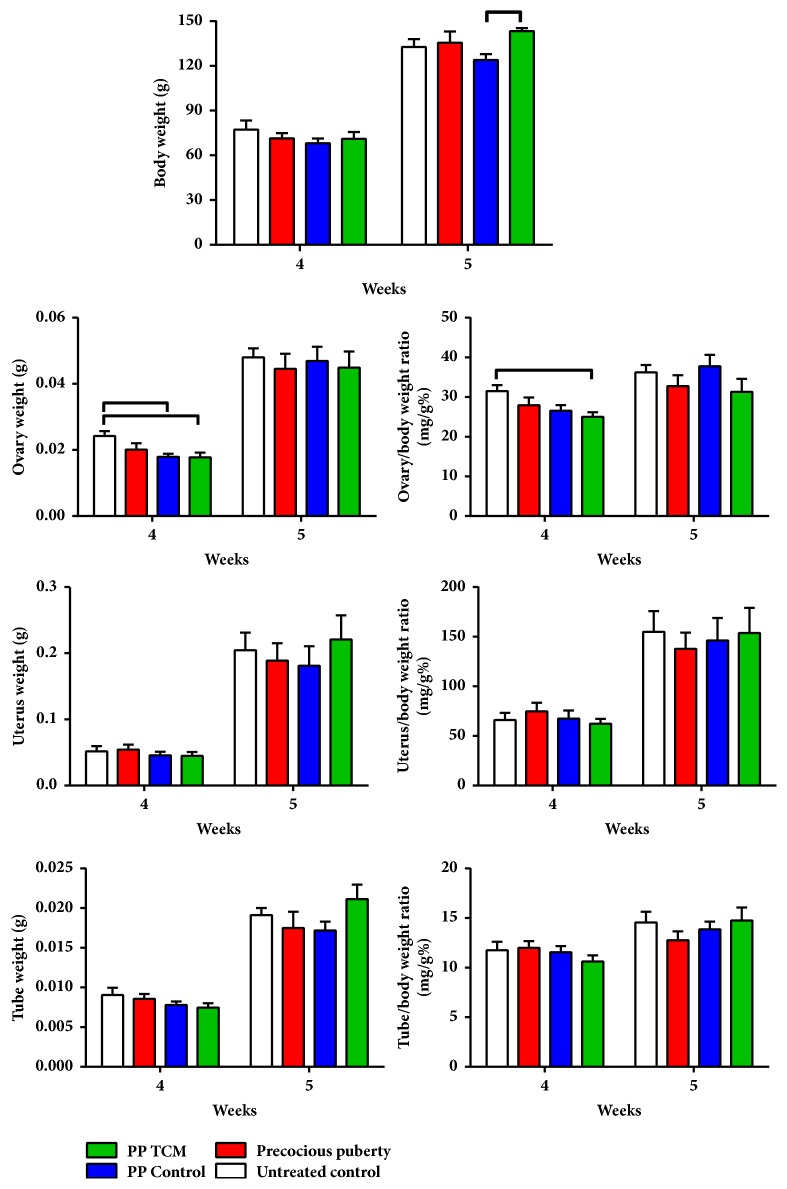
Effects of the nourishing “Yin” and purging “Fire” TCM mixture on body weight and reproductive organ weight in female rats (n = 6). Lines above the columns show significant differences between groups (p < 0.05).

On the day of puberty onset, the net and coefficient weights of the ovary in the PP + TCM group were decreased more significantly than those of the PP model and PP control groups compared to untreated controls (p < 0.05, Figure 4). The net ovary weight in the PP control group was also significantly decreased on the day of puberty onset compared with the untreated controls (p < 0.05). The net and coefficient weights of the uteri and uterine tubes showed no significant differences among the four groups on the day of puberty onset (p > 0.05). On the day of puberty, treatment of precocious puberty with the TCM mixture increased body weight compared with the PP control group (p < 0.05, Figure 4). On the day of puberty onset, the mean ovary weight in the PP + TCM group had a tendency to decrease compared to the other groups, although these differences were not statistically significant (p > 0.05). In addition, the mean weights of the uteri and ovarian tubes in the PP + TCM group tended to be greater compared to the other groups on the day of puberty onset, but these differences were also not significantly different (p > 0.05).

#### 3.1.3. The TCM Mixture Increased LH and P4 Secretions in a Rat Model of Precocious Puberty

**Figure 5:**
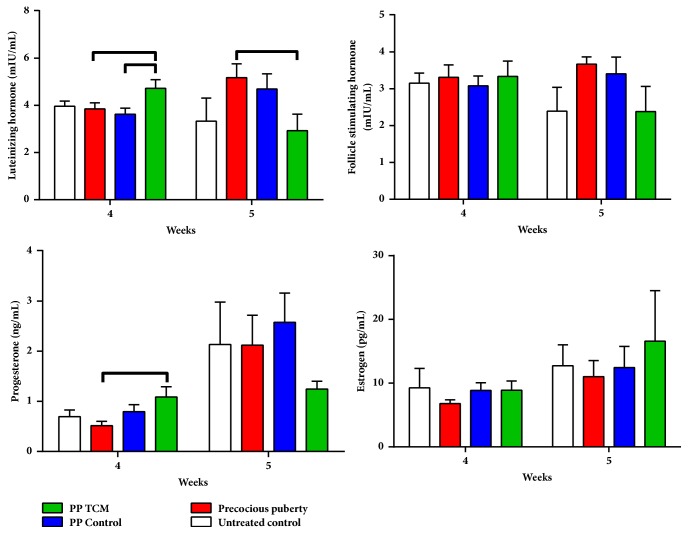
Effects of the nourishing “Yin” and purging “Fire” TCM mixture on sex hormone levels in female rats (n = 6). Lines above the columns show significant differences between groups (p < 0.05).

On the day of puberty onset, serum LH and P4 levels in the PP + TCM groups were higher compared to the other groups (p < 0.05, Figure 5). The serum E2 and FSH levels were not significantly different in any group on the day of puberty onset (p > 0.05). On the day of puberty, treatment of precocious puberty with the TCM mixture had decreased LH concentrations compared to the PP model group (p < 0.05, Figure 5). These differences were not observed in other hormones on the day of puberty onset (p > 0.05).

#### 3.1.4. The TCM Mixture Altered the Lin28/Let7 Pathway in a Rat Model of Precocious Puberty

**Figure 6:**
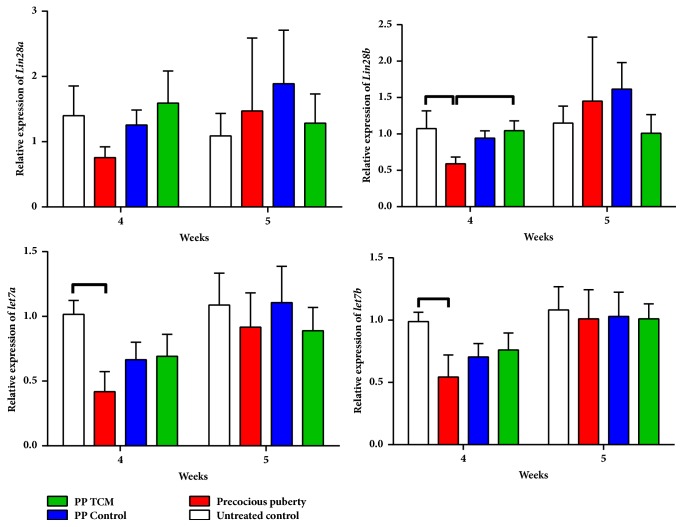
Effects of the nourishing “Yin” and purging “Fire” TCM mixture on the expression of* Lin28a* and* Lin28b* mRNAs and* let7a* and* let7b* miRNAs in the hypothalamus in female rats (n = 6). Lines above the columns show significant differences between groups (p < 0.05).

Induction of precocious puberty decreased the expressions of* let7a* and* let7b* on the day of puberty onset (p < 0.05, Figure 6). On the day of puberty onset, hypothalamic expression of* Lin28b* in the PP + TCM group was greater than in the PP model group (p < 0.05). In addition, the TCM mixture had the same effect on* Lin28a* expression on the day of puberty onset. On the day of puberty, changes in the mRNA and miRNA expression levels were no longer significant.

### 3.2. Experiment 2: Overexpression of* Lin28a* by Hypothalamic Lentivirus Injection

#### 3.2.1. Overexpression of* Lin28a* Delayed Vaginal Opening

**Figure 7:**
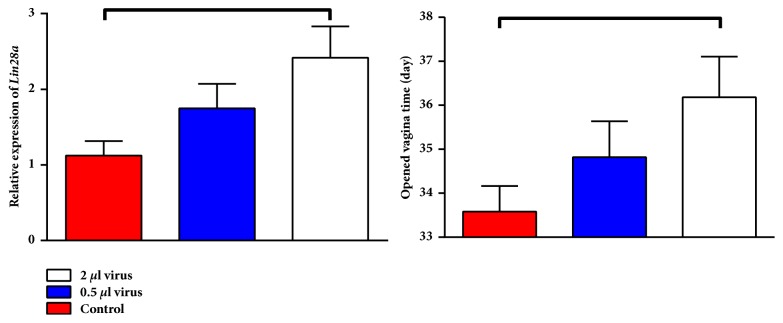
Effects of overexpression of* Lin28a* in the hypothalamic arcuate nucleus on the relative expression of* Lin28a* and the vaginal opening time in three groups in female rats (n = 6). Lines above the columns show significant differences between groups (p < 0.05).

The induction of overexpression of* Lin28a* decreased the number of rats with a fully opened vagina on the day of puberty onset (p < 0.05, Figure 7). This effect was dose dependent and increased concentrations of lentivirus and thus increased* Lin28a* overexpression which decreased the number of rats with a fully opened vagina on the day of puberty onset (p < 0.05).

#### 3.2.2. Overexpression of* Lin28a* Decreased Ovary Weight

**Figure 8:**
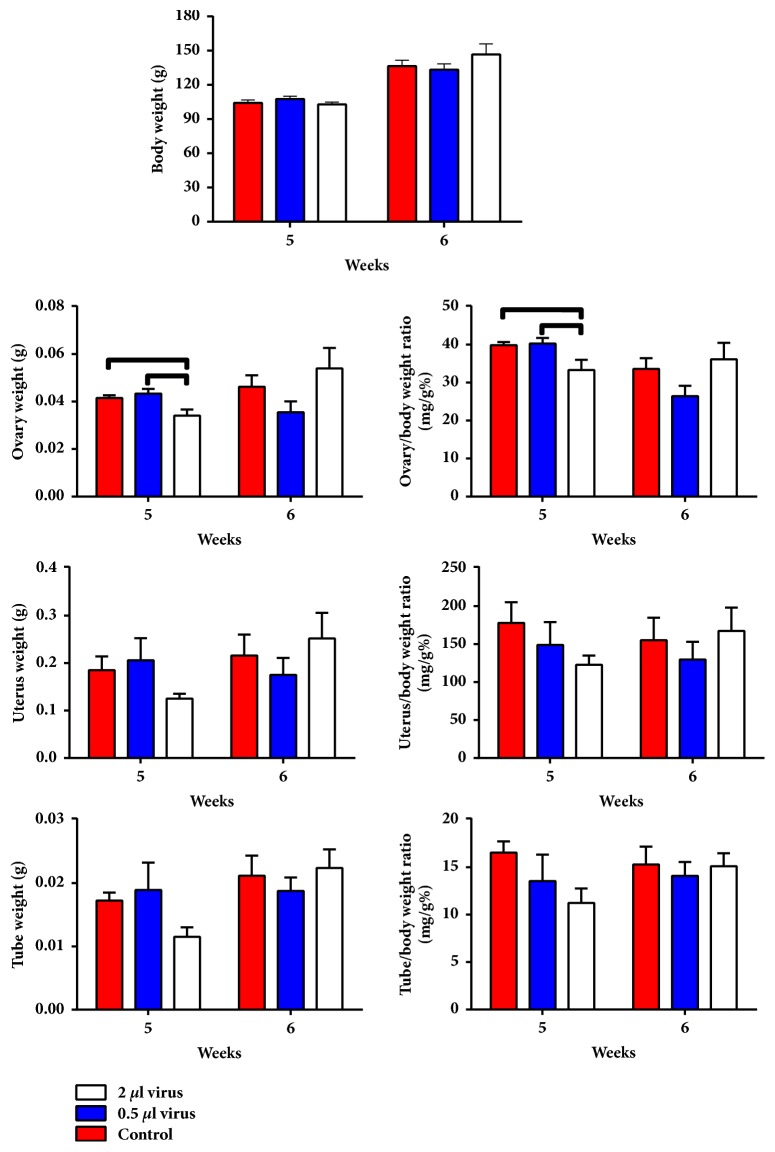
Effects of overexpression of* Lin28a* specifically in the hypothalamic arcuate nucleus on body weight and reproductive organ weights in female rats. Lines above the columns show significant differences between groups (p < 0.05).

The net and coefficient weights of the ovary in the* Lin28a* overexpression group were decreased compared with the controls (p < 0.05, Figure 8). Body weight and the net and coefficient weights of the uteri and uterine tubes showed no significant differences in any of the three groups on the day neither of puberty nor after puberty (p > 0.05, Figure 8).

#### 3.2.3. Overexpression of* Lin28a* Did Not Significantly Alter Hormone Secretions

**Figure 9:**
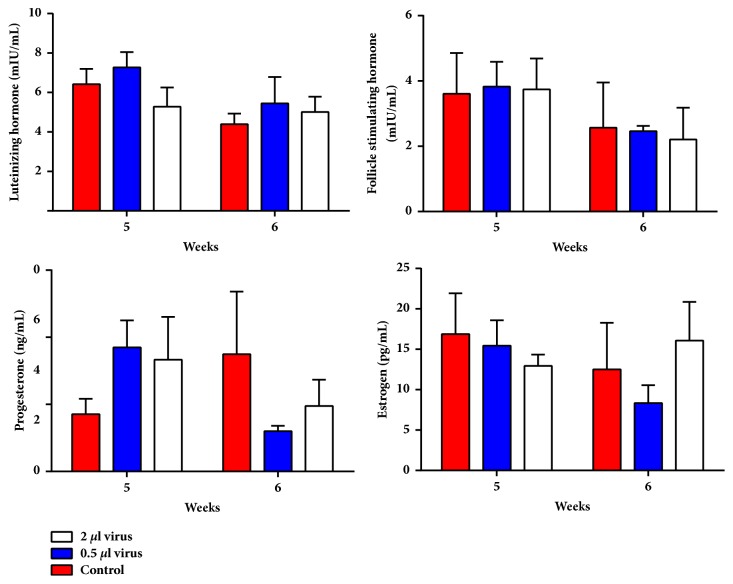
Effects of overexpression of* Lin28a* in the hypothalamic arcuate nucleus on sex hormones in female rats (n = 6). No significant changes were observed in any of the groups.

The mean serum LH and P4 levels were not significantly altered by overexpression of* Lin28a* (p > 0.05, Figure 9). The serum levels of other hormones after overexpression of* Lin28a* in all groups were not significantly different on the day of puberty or after puberty (p > 0.05).

## 4. Discussion

Retrospective studies have demonstrated the early onset of puberty in girls even before the age of 8 years, and early breast development and earlier menarche are the signs of precocious puberty in girls [1]. Precocious puberty has adverse effect on body growth and the psychosocial life of the affected girls [50]. In addition, there is increased risk of metabolic diseases in relation to precocious puberty, and this confirms the importance of developing preventive measures [51, 52]. In 2009, a genome-wide association analysis performed by four research groups found that some SNPs in or near the* LIN28B* gene were related to the age of menarche in girls [21–23, 53]. On the most recent study, there are over 380 loci associated with age at menarche in ore near* LIN28B l*ocus [28]. With a more extensive meta-analysis, the previous observations were confirmed, and it was shown that there is a correlation between* Lin28B* expression and body mass index, breast development, and adult height, suggesting that* Lin28B* is associated with age at menarche and puberty onset [54]. In addition, the largest and most comprehensive paper on the health effects of later puberty was performed by Day et al. in 2015 based on 250,037 women in the UK Biobank, where they showed the effects of* LIN28B* in humans are similar in both sexes and have implications for postnatal growth, pubertal growth, final height, cancer risk, etc. In this study, they also performed the largest scale assessment to date of the potential impact of puberty timing on risks of adverse health outcomes. And the associations between early puberty timing and type 2 diabetes/cardiovascular disease in women were robustly confirmed which could implicate new links between puberty timing in both men and women and a wide range of health outcomes [55]. The meta-analysis also identified some proteins encoded by the* INHBA* and* PCSK2* loci (in or near LIN28B) that affected the release of FSH and LH [54], and this supports the hypothesis that the Lin28/let7 signaling pathway regulates pubertal development.

Lin28 regulates the timing of development of* C. elegans* [9, 15, 56]. Mutations in the heterochronic gene* lin28* of* C. elegans*, which decrease the expression of Lin28, cause precocious development in which diverse events specific to the second larval stage are skipped. Conversely,* C. elegans* shows delayed development when the expression of Lin28 is increased [9, 15, 56]. A transgenic mouse model overexpressing Lin28a to study the relationship between the Lin28 signaling pathway and the timing of puberty onset showed that, compared with littermate pups, the timing of vaginal opening, the time of first estrus, and the age of first pregnancy in the transgenic female mice were all delayed [17]. Subsequent mouse study has also showed the possible effects of* Lin28a* on puberty and growth by overexpression and knockdown in the mouse model. In 2016, Corre et al. evaluated* Lin28b* loss-of-function (LOF) mice and Lin28a gain-of-function (GOF) mice and found that, with the timing of puberty was assessed by vaginal opening (VO) and preputial separation (PS), male* Lin28b *LOF and male* let7* GOF mice displayed alteration of pubertal timing, with later PS than controls. In contrast, both male and female* Lin28a* GOF mice displayed late onset of puberty. The results pointed toward a complex system of regulation by* Lin28a, Lin28b*, and* let7*, in which* Lin28b* and* let7 *can impact both puberty and growth in a sex-specific manner, raising the possibility that this pathway may contribute to differential regulation of male and female growth and puberty in humans [19].

These studies showed that puberty onset might occur relative to the downregulated expression of Lin28, and thus we hypothesized that overexpression of Lin28 only in the hypothalamus leads to a delay in puberty onset.

There is much evidence to support a role for the Lin28/let7 signaling pathway in the onset of puberty. There is a double-negative feedback loop between Lin28 and let7 [11–13]. The hypothalamic ARC has a close relationship with puberty onset [20], and* Lin28* and* Lin28b* mRNAs are mainly expressed in reproductive tissues, including the hypothalamus [47]. In addition, a recent study showed that seven of the eight miRNAs of the let7 family are highly expressed in the hypothalamic ARC and paraventricular nuclei in adult rats [57]. To clarify the role of the Lin28/let7 signaling pathway in the HPG axis, Experiment 2 in the present work used the injection of lentiviral vectors into one side of the hypothalamic ARC to overexpress* Lin28a* and thus upregulate the Lin28/let7 signaling pathway. To monitor puberty development in the female rats, we evaluated the vaginal opening time, the serum sex hormone levels, and the uterine and ovary weights. The relative expression level of* Lin28a* was significantly greater in the high-dose group compared to the control group, and this demonstrated that the lentivirus vectors had been efficiently transfected into the hypothalamus and that the expression of the* Lin28a* gene was successfully upregulated. However, with the overexpression* Lin28a* in rats, although the hormone did not have major difference with P value, from the trend of LH, E2, and P4, we could found that they had opposite trend in 5 weeks and 6 weeks between 2 ul virus group and control group. It can indirectly suggest that the puberty time of two groups was different. In particular the hormones of LH of control group are higher compared to the 2ul group; combining the time delay of VO, it can be indirectly demonstrated that timings of puberty of 2 ul virus group and control group are not the same. In addition, the hormone secretion such like FSH always has a range during a day and has a peak of secretion. Before the end of the luteal phase, there is a slight rise in FSH that seems to be of importance to start the next ovulatory cycle [58, 59]. So it was possible that we might not collect the different hormone at the same peak of secretion in all rats. However, it would be better to take more than two time points for taking samples in the further study.

On the day of puberty onset, we showed that hypothalamic expression of* Lin28a* and* Lin28b* in the PP + TCM group was the same as in untreated controls, and they also had the same timing of vaginal opening. In our study, induction of* Lin28a* overexpression in the hypothalamic ARC decreased the number of rats with opened vagina on the day of puberty, indicating that overexpression of* Lin*28a in the hypothalamic ARC and upregulation of the Lin28/let7 signaling pathway can lead to delayed puberty onset in rats. These results further strengthen the hypothesis that the Lin28/let7 signaling pathway plays an important role in regulating puberty onset. In the hypothalamus, the ARC nuclei are important in the modulation of neuroendocrine function and are closely related to the regulation of GnRH neurons [60, 61]. Consistent with this,* Lin28a* transgenic mice show the same precocious puberty phenotype as in humans [17]. Furthermore, our findings show that induction of precocious puberty decreases the expression of* let7a* and* let7b* on the day of puberty onset. Similar to our findings, it has been shown that* let7a* and* let7b* miRNAs control the timing of major developmental events in* C. elegans* and that loss-of-function mutations result in precocious development [9].

In a previous study, the nourishing “Yin” and purging “Fire” TCM herb mixture was shown to delay the onset of puberty, increase the final height in adulthood, and delay the age of menarche in girls [38]. However, the precise mechanism for how this TCM mixture exerts its effects is still unclear. Our study is the first to explore the effects of the nourishing “Yin” and purging “Fire” TCM mixture on the Lin28/let7 signaling pathway in the hypothalamus and the timing of puberty. The Lin28/let7 signaling pathway has previously been shown to play a critical role in puberty development [6]. S. Sangiao et al. found that* Lin28b* mRNA abundance declines from high levels during the infantile period to low values at the time of puberty in rats;* let7a or let7b* miRNA levels were minimal neonatally and progressively increased during postnatal maturation in both male and female rats. Furthermore, in 2016, the study of neonatal expression of* Lin28a* and* Lin28b* showed it was low and rose markedly during the infantile period; yet, expression patterns diverged thereafter, with persistently elevated levels only for* Lin28b*, which peaked at puberty.* Lin28a, let7a, *and* let7b *showed profiles opposite to* Lin28b*. After the puberty, the expressions of them are much lower than before.* Lin28a *and* Lin28b* mRNAs displayed low expression during the neonatal period, increasing markedly during infantile period. However, their expression profiles diverged thereafter, so that* Lin28a* mRNA levels showed a subsequent decrease to adulthood, while* Lin28b* mRNA levels remained high from infantile to adulthood, with peak expression around the time of puberty.* Let7a *and* let7b* showed expression profiles that were grossly opposite to those of* Lin28b*. All of them declined in expression after the neonatal/infantile period. However, while* let7a *decreased sharply after birth,* let7b* increased between the neonatal and infantile age, to decline thereafter until puberty [18, 62], while, in our study, we started to gavage the rats with the TCM mixture until Day 15 after birth, and the time points of sampling collection were 4 weeks and 5 weeks. At these two time points,* Lin28a, let7a, *and* let7b* had already showed low expression. The TCM mixture might have the effect on the expression of* Lin28a, let7a, *and* let7b*. However, during the puberty, it should be so difficult to detect the difference in groups on this low expression of* Lin28a, let7a, and let7b*. Compared with the PP group, the effects of TCM mixture (PP+TCM group) on* Lin28a*,* let7a, *and* let7b* were not significantly different. The only significant difference existed only in* Lin28b.* Thus, both* Lin28a* and* Lin28b* and their related miRNAs* let7a* and* let7b* likely have a role in pubertal development, and the expression levels* of Lin28b* of the pathway were affected by the nourishing “Yin” and purging “Fire” TCM herb mixture.

The vaginal opening in rats treated with the TCM mixture was delayed, and at the onset of puberty the mean ovarian weight in the PP + TCM group was lower compared to the untreated controls. Similar to our finding, the nourishing “Yin” and purging “Fire” TCM herb mixture delayed vaginal opening in female rats in the precocious puberty model [4, 5]. Thus we conclude that the nourishing “Yin” and purging “Fire” TCM herb mixture can delay puberty onset in female precocious puberty rats.

Because body weight has an impact on puberty onset and vice versa [63], the effect of the TCM mixture on body weight was evaluated in our study. We showed that, on the day of puberty, the body weight had increased in the PP + TCM group compared to the PP control group and was the same as the untreated controls. The TCM mixture only had a mild positive effect on body weight, which was consistent with the study of Zeng* et al*. [5] in a rat model of precocious puberty.

Because alterations of sex-related hormone concentrations affect the process of puberty, we evaluated the serum hormone levels of LH, FSH, E2, and P4. On the day of puberty onset, serum LH and P4 levels in the PP + TCM group were higher than the other groups, but, on the day of puberty, treatment of precocious puberty with the TCM mixture maintained LH and P4 levels at lower levels than the other groups. Similar to our findings, a previous study showed that rats at puberty were still at an early stage of puberty after treatment with the nourishing “Yin” purging “Fire” TCM herb mixture and that hormone levels were low [5].

Treatment with the nourishing “Yin” purging “Fire” TCM herb mixture in a rat model of PP increased LH and P4 at week 4. The TCM was made from different herbs in which* Rehmannia glutinosa* has the highest concentration and it has phytoprogestogenic effects on the hypothalamic-pituitary-adrenal axis [64, 65]. In addition,* Rehmannia glutinosa* has shown ability to mimic the progesterone stimulating actions on osteoblasts and inhibitions of osteoclastic activity [66]. Furthermore, catalpol is also a compound that has been separated from* Rehmannia glutinosa* and* Scrophularia buergeriana* [67], the other available ingredient in our recipe that has P4 and LH stimulatory effects. The adjustment of catalpol separated from* Rehmannia glutinosa* over P4 and LH is superior to that of 17*β*-E2 in the sera of aged senile rats [68]. On the other hand, on the day of puberty at week 5, the effects of TCM on LH and P4 can be explained via the anti-GnRH effects of* Anemarrhena asphodeloides* and* Cortex phellodendri* on the activated GnRH neurons after puberty.* Anemarrhena asphodeloides* and* Cortex phellodendri* inhibited the GnRH mRNA expression in GT1-7 cells [69]. Therefore, the TCM mixture might have inhibitory effects on sex-related hormone levels during the onset of puberty.

In conclusion, the nourishing “Yin” and purging “Fire” TCM herb mixture delayed the onset of puberty in female rats and upregulated the expression of hypothalamic* Lin28b* mRNA on the day of puberty onset. In addition, overexpression of* Lin28a* specifically in the hypothalamus increased the number of rats with complete vaginal opening on the day of puberty onset. Our findings suggest that downregulation of* Lin28a* and* Lin28b* accelerate the normal onset of puberty, and the mechanism behind this effect on precocious puberty is likely related to the hypothalamic Lin28/let7 signaling pathway.

## Figures and Tables

**Table 1:** Ingredients (in grams per 1000 ml of water) of the nourishing “Yin” and purging “Fire” Traditional Chinese Medicine herbal mixture for the treatment of precocious puberty in rats.

Scientific name	Chinese name	Common name	Family	Weight
*Rehmannia glutinosa*	Sheng Di huang	Rehmannia root	Scrophulariaceae	15g
*Scrophularia buergeriana*	Xuan shen	Buerger's Figwort	Scrophulariaceae	9g
*Anemarrhena asphodeloides*	Zhi mu	Zhimu	Liliaceae	9g
*Cortex phellodendri*	Huang bai	Phellodendron bark	Rutaceae	9g
*Paeonia suffruticosa* Andr.	Dan pi	Moutan	Ranunculaceae	9g
*Alisma plantago-aquatica* L. var. orientale Sam.	Ze xie	Alisma oriental	Alismataceae	9g
*Prunella vulgaris* L.	Xia ku cao	Common self-healing	Lamiaceae	9g
*Chinemys reevesii* (Carapax et Plastrum Testudinis)	Gui jia	Plastron of fresh-water tortoise	Testudinidae	12g
*Hordeum vulgare* L.	Mai ya	Barely	Gramineae	30g
*Gentiana scabra* Bge	Long Dan Cao	Chinese gentian	Gentianaceae	6g

**Table 2:** Sequences of the real-time PCR primers for evaluation of the relative expression of the *Lin28a*, *Lin28b*, *GAPDH*, *mir-let7a*, *mir-let7b*, and *U6* genes in the rat.

Primer	Sequence	Amplicon length (bp)
Lin28a-F	AGTACCCTGCCACTGAGTTAT	132
Lin28a-R	GGAAGCCAAGATTGTGAA	
Lin28b-F	AGAAGTGCTGCCTTGCCTTA	73
Lin28b-R	TTGGGTGACACTCTGATTCGT	
GAPDH-F	ACTTTGGCATCGTGGAAGGG	128
GAPDH-R	TGCAGGGATGATGTTCTGGG	
mir-let7a-F	ACACTCCAGCTGGGTGAGGTAGTAGGTTGTAT	63
mir-let7a-R	TGTCGTGGAGTCGGCAATTC	
mir-let7b-F	ACACTCCAGCTGGGTGAGGTAGTAGGTTGTGT	63
mir-let7b-R	TGTCGTGGAGTCGGCAATTC	
U6-RT	CGCTTCACGAATTTGCGTGTCAT	
U6-F	GCTTCGGCAGCACATATACTAAAAT	95
U6-R	CGCTTCACGAATTTGCGTGTCAT	

**Table 3:** Sequences of the *Lin28a* genes in the lentiviral overexpression experiment.

Construct	Sequence
Lin28a-F	CMV-F CGCAAATGGGCGGTAGGCGTG
Lin28a-R	WPRE-R CATAGCGTAAAAGGAGCAACA

## Data Availability

All data generated or analyzed during this study are included within the article.

## Abbreviations

ARC: Arcuate nucleus
*C. elegans*: * Caenorhabditis elegans*
E2: Estrogen
FSH: Follicle-stimulating hormone
GnRH: Gonadotropin releasing hormone
HPG: Hypothalamus-pituitary-gonadal
LH: Luteinizing hormone
miRNAs: MicroRNAs
P4: Progesterone
PP: Precocious puberty
TCM: Traditional Chinese Medicine.
